# Impact of disease activity patterns on health-related quality of life (HRQoL) in patients with systemic lupus erythematosus (SLE)

**DOI:** 10.1136/lupus-2024-001202

**Published:** 2024-07-29

**Authors:** Elena Elefante, Luca Gualtieri, Davide Schilirò, Chiara Stagnaro, Viola Signorini, Dina Zucchi, Chiara Cardelli, Linda Carli, Francesco Ferro, Chiara Tani, Marta Mosca

**Affiliations:** 1 Rheumatology Unit, University of Pisa, Pisa, Italy

**Keywords:** Systemic Lupus Erythematosus, Patient Reported Outcome Measures, Quality of LIfe

## Abstract

**Objective:**

To assess the impact of different disease activity patterns—long quiescent (LQ), chronically active (CA) and relapsing-remitting (RR)—on health-related quality of life (HRQoL) in a cohort of patients with systemic lupus erythematosus (SLE).

**Methods:**

A retrospective, monocentric analysis of prospectively collected data. Adult SLE outpatients were enrolled between 2017 and 2021.

For each year of follow-up, three disease activity patterns were defined: LQ if at each visit clinical Safety of Estrogens in Lupus Erythematosus National Assessment-Systemic Lupus Activity Index (SELENA-SLEDAI)=0, Physician Global Assessment (PGA)=0; CA if at each visit clinical SELENA-SLEDAI >0, PGA >0; RR if patients presented active disease in at least one visit during the observation period, interspersed with periods of remission. These patterns were applied to the year and the 3 years before enrolment.

At enrolment, each patient completed: Short Form 36 (SF-36), Lupus Impact Tracker, Functional Assessment of Chronic Illness Therapy (FACIT), Hospital Anxiety and Depression Scale (HADS). The correlation between disease patterns and Patient-Reported Outcomes was analysed.

**Results:**

241 SLE patients were enrolled, of which 222 had complete clinical data for the 3-year period before enrolment. Both in the year and during the 3 years before enrolment, the most frequent disease pattern was the LQ (154/241 and 122/222 patients, respectively), followed by RR (53/241 and 92/222 patients, respectively) and CA (34/241 and 8/222 patients, respectively).

At baseline, fibromyalgia, organ damage, age and daily glucocorticoid dose were associated with worse HRQoL.

At the multivariable analysis, after adjusting for confounding factors, patients with LQ disease during the 3 years before enrolment presented a better physical HRQoL (SF-36 physical component summary, regression coefficient=3.2, 95% CI 0.51–5.89, p=0.02) and minor depressive symptoms (HADS-D, regression coefficient=−1.17, 95% CI −2.38 to 0.0.27, p=0.055), compared with patients with CA/RR disease.

**Conclusion:**

A persistently quiescent disease may have a positive impact on patients’ physical HRQoL and on depressive symptoms. However, this condition appears insufficient to obtain a significant improvement in mental health, fatigue and disease burden among patients with SLE.

**WHAT IS ALREADY KNOWN ON THIS TOPIC**

- SLE is a complex systemic disease with an unpredictable course. Therefore, global indices of disease activity and damage are often weak predictors of patients’ health-related quality of life (HRQoL) and disease burden.

**WHAT THIS STUDY ADDS**

- In this study, we demonstrate that a long quiescent disease course, for at least 3 years, may have a positive impact on the physical aspects of HRQoL and on depressive symptoms in SLE patients. However, a stable well-controlled disease is still insufficient to improve mental health, social functioning, fatigue and perception of disease burden among patients with SLE.

**HOW THIS STUDY MIGHT AFFECT RESEARCH, PRACTICE OR POLICY**

- This study underlines that it is necessary to find a way to integrate the patient and the physician’s perspectives on the disease into a novel strategy for the management of SLE, in order to improve the communication between the parts promoting patients’ empowerment.

## Introduction

 Systemic lupus erythematosus (SLE) is a chronic, complex disease with an unpredictable course and a wide range of manifestations and organ involvement.^1^

In the last decades, better control of disease activity has been associated with the improvement of long-term prognosis of patients with SLE, in terms of damage accrual, hospitalisations, comorbidities and mortality.^2^

However, this has not been paralleled by a similar improvement in patients’ Health-Related Quality of Life (HRQoL). HRQoL of SLE patients is consistently lower not only when compared with that of matched healthy subjects,^3^ but also when compared with patients with other chronic diseases.^4^,^6^

The relationship between disease activity, organ damage and HRQoL in SLE is controversial and using global activity and damage indices as predictors of quality of life can hinder the correct evaluation of the impact of the disease on patient’s life.^7^

Several studies have demonstrated that the correlation between SLEDAI and SLICC-Damage Index with Patient-Reported Outcomes (PROs) is often weak.^8^,^10^

Actually, global disease activity and damage indices give more weight to the items relative to the most severe disease manifestations, like renal and neuropsychiatric ones, which are the major concerns in the management of SLE from the physician’s point of view. On the other hand, patients’ major concerns are relative to symptoms like pain and fatigue and to the degree of functioning in their daily living.^11^,^13^

In fact, musculoskeletal manifestations in SLE seem to have a stronger association with PROs results, particularly with the physical aspects of HRQoL.^14 15^

Just to make the picture even more complex, HRQoL is often investigated by generic PROs that may not be able to explore all aspects that are relevant for SLE patients, whereas disease-specific questionnaires seem to be more sensitive to changes in disease activity.^10^

However, some data in the literature show that in the life of a patient with SLE, a link exists between the course of the disease and the patient’s perception of health status. Some studies seem to demonstrate that patients with a stable well-controlled disease for a long period of time have a better HRQoL, particularly in the physical domains.^16 17^

So, our hypothesis is that in such a complex disease, with an unpredictable course and a great variety of clinical manifestations, the course of the disease over time, rather than disease activity in a single moment, could have a more significant impact on patients’ HRQoL.

Barr *et al*^18^ have historically defined three main different courses of the disease over time: a relapsing-remitting (RR) course, a long quiescent (LQ) and a chronically active (CA) disease.

Therefore, the goal of this study is to assess the impact of the three different patterns of disease activity on HRQoL, evaluated by both generic and disease-specific PROs, in a monocentric cohort of SLE patients.

## Methods

This is a retrospective, monocentric analysis of prospectively collected data of adult, consecutive SLE outpatients fulfilling the 1997 ACR classification criteria,^19^ or 2012 SLICC classification criteria,^20^ or 2019 EULAR/ACR classification criteria,^21^ regularly followed at the Rheumatology Unit of Pisa. Patients were enrolled in this study in the period between 2017 and 2021.

For each patient, the following data were retrieved from clinical records: demographics, disease duration, cumulative and active organ involvement, organ damage, laboratory data, comorbidities, and treatment. At each visit, disease activity was assessed by using the Safety of Estrogens in Lupus Erythematosus National Assessment-Systemic Lupus Activity Index (SELENA-SLEDAI)^22^ and the Physician Global Assessment (PGA) on a 3 cm visual analogue scale (0 no disease activity, 1 mild, 2 moderate, 3 severe active disease, with decimal values allowed).

Disease status was defined according to the DORIS definition of remission^23^ and the Lupus Low Disease Activity State (LLDAS) definition.^24^ Organ damage was assessed yearly by the Systemic Lupus International Collaborating Clinics Damage Index (SLICC-DI).^25^

For each year of patients’ follow-up, three different disease patterns were defined based on disease activity status: LQ if the disease remained clinically quiescent at each visit (clinical SELENA-SLEDAI=0, PGA=0); CA if the disease was persistently active at each visit (clinical SELENA-SLEDAI >0 and PGA >0); RR if patients presented active disease in at least one visit during the observation period, interspersed with periods of remission.

These patterns were applied to the year before enrolment and the period of 3 years before enrolment.

Patients with at least 1 year of follow-up and at least two visits per year were enrolled.

We excluded patients with a major clinical event/hospitalisation not SLE-related during the study period that could influence their health status.

The prevalence of the different disease activity patterns in the study cohort was evaluated.

Moreover, at enrolment, each patient completed the following PROs:

- The Medical Outcomes Study Short Form 36 (SF-36) questionnaire to assess HRQoL. The questionnaire results are summarised into two global scores: the physical component summary (PCS) and the mental component summary (MCS). Each score ranges from 0 to 100, with higher values representing better self-perceived HRQoL.^26 27^
- The Lupus Impact Tracker (LIT) questionnaire to assess the impact of SLE on daily living which includes 10 questions about cognition, lupus medication, physical health, pain/fatigue impact, emotional health, body image and planning/desires/goals. This questionnaire provides a single summary score from 0 to 100, with lower scores signifying lower impact of SLE on patient life.^28^
- The Functional Assessment of Chronic Illness Therapy (FACIT)-Fatigue questionnaire to assess fatigue. The score ranges from 0 to 52, with lower scores indicating greater fatigue.^29 30^
- The Hospital Anxiety and Depression Scale (HADS) is a 14-item instrument assessing symptoms of anxiety and depression. It consists of two subscales, HADS-D for depression and HADS-A for anxiety. A cut-off of 8 for either subscale indicates a positive screen for anxiety or depression. HADS has been studied in several rheumatic diseases, including SLE.^31^,^33^

The correlation between the different disease patterns (during the year and 3 years before enrolment) and the PROs used was analysed.

The study was approved by the local ethics committee. The name of the ethics committee is ‘Comitato Etico di Area Vasta Nord Ovest’, and the committee’s reference number is 14 478.

### Statistical analysis

Continuous data have been reported as median and IQR or as mean and SD as appropriate. Categorical data have been reported as a percentage. The Student’s t-test was conducted for two group comparisons of PROs. Unadjusted linear regression analysis was also performed to assess associations between baseline continuous variables and PROs. A one-way analysis of variance (ANOVA) with Bonferroni correction was performed for comparison of PROs across the LQ, CA and RR groups. Multivariable analysis has been performed by multiple linear regression. The multiple linear regression model was adjusted for age at enrolment, SLICC-DI, daily glucocorticoid dose and fibromyalgia, which were significantly associated within univariate analysis.

All p values less than 0.05 have been considered statistically significant. Statistical analysis has been performed using STATA V.13 software.

### Patient and public involvement

Patients and the public were not directly involved in the design of this study or in the analysis of data.

## Results

We enrolled 241 consecutive adult outpatients with SLE who were predominantly women (92.3%) and white (96.8%). The mean age at enrolment was 45±12.6 years with a mean 14±10.1 years of disease duration. 222/241 (92.1%) had complete clinical data for the 3-year period before enrollment.

The majority of patients had a history of articular (172/241, 71.4%), cutaneous (151/241, 62.6%) and haematological (127/241, 52.7%) involvement; 42.7% (103/241) had a previous renal involvement.

**Figure 1:**
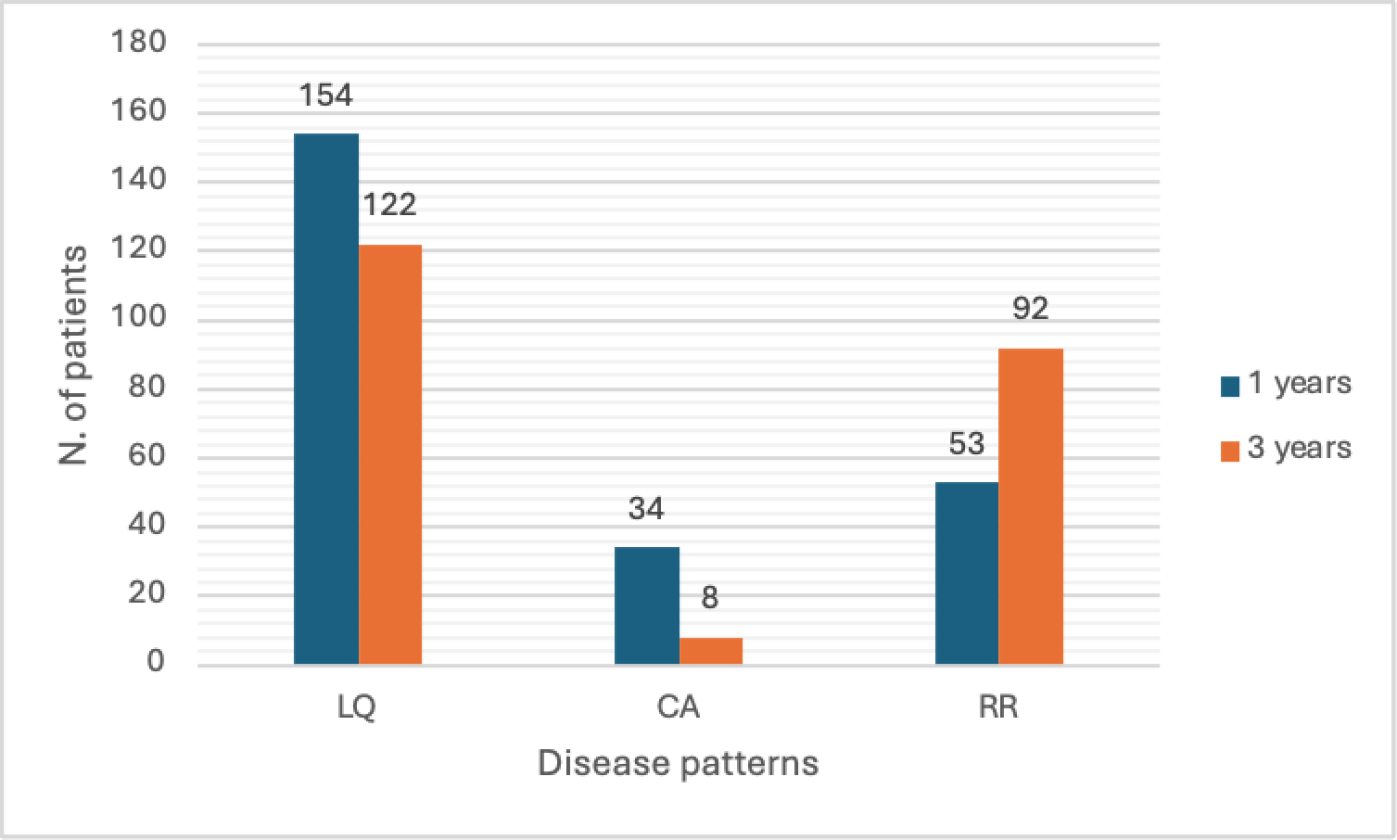
Prevalence of the disease activity patterns in the study cohort.

At enrolment, patients presented an overall low disease activity with a median SELENA-SLEDAI score of 2 (IQR 0–4). The majority of patients enrolled were in remission on or off treatment (174/241, 72.2%), while 10.8% (26/241) were in LLDAS. Almost 15% of patients presented a concomitant fibromyalgia. The most frequent active disease manifestations at enrolment were cutaneous (34/241), followed by haematological (16/241) and articular (12/241); 11 patients had active renal involvement. At enrolment, the distribution of the different disease activity patterns was as follows: LQ 154 (63.9%) and 122 (54.9%) patients, CA 34 (14.1%) and 8 (3.6%) patients, RR 53 (21.7%) and 92 (41.5%) patients, during the previous 1 and 3 years, respectively (figure 1).

The baseline characteristics of patients enrolled are detailed in table 1.

**Table 1:** Baseline characteristics of the study cohort

Baseline characteristics	Entire cohort	LQ1	RR1	CA1
N	241	154	53	34
Female, N (%)	222/241 (92.3%)	147/154 (95.4%)	44/53 (83%)	30/34 (88.2%)
Mean age (years)±SD	45±12.6	46±12.5	41±12.3	42±12.7
White, N (%)	233/241 (96.7%)	150/154 (97.4%)	50/53 (94.3%)	34/34 (100%)
Mean disease duration (years)±SD	14±10.1	16±10.5	12±8.5	10±8.6
Median SELENA-SLEDAI (IQR)	2 (0–4)	0 (0–2)	2 (1–4)	4 (3–6)
Median SLICC (IQR)	0 (0–1)	0 (0–1)	0 (0–1)	0 (0–0)
Fibromyalgia, N (%)	36/241 (14.9%)	23/154 (14.9%)	10/53 (18.9%)	3/34 (8.8%)
LLDAS (no remission), N (%)	26/241 (10.8%)	0	8/53 (15.1%)	10/34 (29.4%)
Remission on or off treatment, N (%)	174/241 (72.2%)	154/154 (100%)	27/53 (50.9%)	0
Mean daily glucocorticoid dose (mg)±SD	2±2.9	1.3±1.7	2.8±2.8	4.4±5.1
Glucocorticoid therapy, N (%)	107/241 (44.4%)	64/154 (41.5%)	33/53 (62.3%)	25/34 (73.5%)
Hydroxychloroquine, N (%)	203/241 (84.2%)	125/154 (81.2%)	45/53 (84.9%)	33/34 (97%)
Immunosuppressant, N (%)	103/241 (42.7%)	58/154 (37.7%)	30/53 (56.6%)	15/34 (44.1%)
bDMARD, N (%)	37 (15.4%)	15/154 (9.7%)	11/53 (20.7%)	13/34 (38.2%)

bDMARDbiologic Disease-Modifying Anti-Rheumatic DrugCA1chronically activeLLDASLupus Low Disease Activity StateLQ1long quiescent diseaseRR1relapsing-remittingSELENA-SLEDAISafety of Estrogens in Lupus Erythematosus National Assessment-Systemic Lupus Activity IndexSLICCSystemic Lupus International Collaborating Clinics

Patients with a CA disease had a mean number of visits of 3.03±1.29 with a mean SELENA-SLEDAI score of 4.97±2.3 (min 2–max 10) and a PGA score of 1±0.34, during the year before enrolment. During the 3 years before enrolment, CA patients underwent to a mean number of 8.33±3.14 visits with a mean SELENA-SLEDAI score of 4.41±2.19 (min 2.2 – max 8.5) and a PGA score of 1.06±0.34. Patients with a RR course had a mean number of visits with active disease of 1.33±0.67 over a total number of visits of 3.19±0.91 during the year before enrolment, and a mean number of visits with active disease of 2.31±1.53 over a mean total number of visits of 7.38±2.1 in the 3 years before enrolment. Mean SELENA-SLEDAI score during visits with active disease was similar during 1 and 3 years before study entry (5.51±2.67 and 5.3±2.47, respectively; min 1–max 16), and a mean PGA score of almost 1 (1.05±0.4 in the year before and 1.04±0.39 in the 3 years before enrolment). These data are summarised in online supplemental table S1.

The results of PROs at enrolment are reported in table 2.

**Table 2:** Results of PROs at enrolment in the study cohort

SF-36 PCS (mean±SD)	47.4±10
SF-36 MCS (mean±SD)	43.7±11.8
FACIT-F (mean±SD)	37.8±10.3
LIT (mean±SD)	26.3±21.3
HADS-anxiety (mean±SD)	6.8±3.9
HADS-depression (mean±SD)	5.4±3.6

First of all, we investigated which clinical characteristics were associated with PROs, at the time of our evaluation.

FACIT-FFunctional Assessment of Chronic Illness Therapy – Fatigue ScaleHADSHospital Anxiety and Depression ScaleLITLupus Impact TrackerPROsPatient-Reported OutcomesSF-36 MCSShort Form 36 mental component summarySF-36 PCSShort Form 36 physical component summary

At univariate analysis, disease activity at enrolment, both when considered as SELENA-SLEDAI score and when considered according to the definitions of LLDAS or remission, was not significantly associated with PROs results. On the contrary, patients with fibromyalgia showed significantly worse PROs results in almost all of the questionnaires used (with p values between p<0.01 and p<0.0001) (table 3). Moreover, although our study cohort presented an overall low organ damage, we found that a higher SLICC-DI score was significantly associated with worse scores in most of the questionnaires used (with the strongest correlation being with SF-36 PCS, FACIT and LIT, p<0.001), even after adjusting for fibromyalgia (data are shown in online supplemental table S2). Finally, age at enrolment and daily glucocorticoid therapy were significantly associated with a worse physical HRQoL, expressed by the SF-36 PCS score (p<0.01). In table 4, the results of the unadjusted linear regression analysis have been reported as regression coefficient (Coef.) and p value.

**Table 3:** Impact of fibromyalgia and LLDAS/remission state at baseline on PROs

	FM yes	FM no	P value	LLDAS/Rem yes	LLDAS/Rem no	P value
SF-36 PCS	41.1±9.5	48.6±9.7	**<0.0001**	47.9±10.2	45.5±9	0.14
SF-36 MCS	38.3±10.5	44.7±11.8	**<0.01**	44.1±12.3	41.9±9.3	0.27
FACIT	30±11.3	39.4±9.4	**<0.0001**	38.1±10.4	36.7±10.2	0.43
LIT	39.4±22	23.7±20.1	**<0.001**	25.4±21.4	30.4±20.7	0.18
HADS-A	8.1±3.8	6.5±3.9	0.07	6.5±4	7.7±3.7	0.1
HADS-D	7.7±3	4.9±3.6	**<0.001**	5.1±3.5	6.3±4	0.08

Bold values represent statistically significant values.

FACITFunctional Assessment of Chronic Illness TherapyFMfibromyalgiaHADS-AHospital Anxiety and Depression Scale for anxietyHADS-DHospital Anxiety and Depression Scale for depressionLITLupus Impact TrackerLLDASLupus Low Disease Activity StatePROsPatient-Reported OutcomesSF-36 MCSShort Form 36 mental component summarySF-36 PCSShort Form 36 physical component summary

**Table 4:** Association of baseline continuous characteristics with PROs

	SLICC-DI	Age at enrolment	GC daily dose	Disease duration	SELENA-SLEDAI
SF-36 PCS	Coef −0.04**p=0.000**	Coef −0.15**p=0.003**	Coef −0.05**p=0.004**	Coef −0.06p=0.33	Coef −0.01p=0.30
SF-36 MCS	Coef −0.01p=0.1	Coef −0.11p=0.07	Coef −0.01p=0.35	Coef −0.06p=0.26	Coef .002p=0.88
FACIT	Coef −0.04**p=0.000**	Coef −0.08p=0.15	Coef −0.03p=0.16	Coef 0.04p=0.52	Coef −0.01p=0.61
LIT	Coef 0.02**p=0.000**	Coef 0.067p=0.58	Coef 0.015p=0.13	Coef −0.03p=0.29	Coef 0.006p=0.39
HADS-A	Coef 0.06**p=0.03**	Coef 0.03p=0.20	Coef 0.099p=0.14	Coef 0.28p=0.20	Coef −0.04p=0.45
HADS-D	Coef 0.08**p=0.003**	Coef 0.04p=0.10	Coef 0.05p=0.46	Coef 0.25p=0.29	Coef −0.01p=0.79

Bold values represent statistically significant values.

CoefcoefficientFACITFunctional Assessment of Chronic Illness TherapyGCglucocorticoidsHADS-AHospital Anxiety and Depression Scale for anxietyHADS-DHospital Anxiety and Depression Scale for depressionLITLupus Impact TrackerPROsPatient-Reported OutcomesSELENA-SLEDAISafety of Estrogens in Lupus Erythematosus National Assessment-Systemic Lupus Activity IndexSF-36 MCSShort Form 36 mental component summarySF-36 PCSShort Form 36 physical component summarySLICCSystemic Lupus International Collaborating Clinics

We then analysed the results of PROs according to the disease activity pattern, by comparing LQ patients with a CA or a RR course, during 1 and 3 years before enrolment. As reported in table 5, at the univariate analysis, as far as the disease activity pattern during the year before enrolment is concerned, an LQ disease (LQ1) seems to be associated with a trend for a better physical HRQoL, lower fatigue and lower burden of the disease, but no questionnaires reached the statistical significance. We also analysed the CA1 and RR1 groups during the year before enrolment separately, and we did not find any significant difference in the questionnaire results across groups. However, an LQ disease during the 3 years before enrolment (LQ3) resulted significantly associated with a better physical HRQoL (SF-36 PCS) and a tendency for minor depressive symptoms (HADS-D).

**Table 5:** Results of PROs according to the disease activity patterns at the univariate analysis

	LQ 1 year	CA/RR 1 year	P value	LQ 3 years	CA/RR 3 years	P value
SF-36 PCS	48.4±10.4	45.9±9	0.06	48.8±10.7	45.9±9.4	**0.04**
SF-36 MCS	44.4±12.1	42.5±11.1	0.22	44.2±12.9	43.5±10.7	0.67
FACIT	38.9±9.6	36.3±11.2	0.08	38.9±9.9	36.7±11.1	0.16
LIT	24.3±20.5	29.5±22.3	0.09	24.9±20.9	27.6±22.4	0.39
HADS-A	6.6±4	7±3.9	0.56	6.3±4	7±3.9	0.33
HADS-D	5.1±3.6	5.8±3.7	0.29	4.8±3.5	5.9±3.7	0.09

Bold values represent statistically significant values.

CAchronically activeFACITFunctional Assessment of Chronic Illness TherapyHADS-AHospital Anxiety and Depression Scale for anxietyHADS-DHospital Anxiety and Depression Scale for depressionLITLupus Impact TrackerLQlong quiescentPROsPatient-Reported OutcomesRRrelapsing-remittingSF-36 MCSShort Form 36 mental component summarySF-36 PCSShort Form 36 physical component summary

Importantly, at the multivariable analysis, considering the disease pattern during the 3 years before enrolment and after adjusting for the other factors influencing the PROs results (age at enrolment, SLICC-DI, daily glucocorticoid dose and fibromyalgia), we confirmed that patients with a LQ disease presented a better physical HRQoL (SF-36 PCS, p=0.02, Coef 3.2, 95% CI .51 to 5.89) and a tendency for minor depressive symptoms (HADS-D, p=0.055, Coef −1.17, 95% CI −2.38 to 0.0.27), compared with patients with CA or RR disease course.

## Discussion

The main objective of this study was to investigate if disease activity patterns may have an impact on patient’s perception of health status and HRQoL.

We evaluated the pattern of disease activity only in the period (of 1 or 3 years) immediately preceding the enrolment in the study, in order to analyse the impact of the course of the disease in the last period on patients’ quality of life.

In this analysis, we found that almost half of the patients presented an LQ disease course even during a follow-up of three consecutive years.

During a 3-year follow-up, as expected, the percentage of patients with a RR disease increased compared with the 1-year follow-up only (41.5% vs 21.7%).

Previous studies evaluated the frequency of different disease patterns in SLE. Differently from our study, in 1999, Barr *et al* showed that the CA was the most frequent one in the Hopkins Lupus Cohort, followed by the RR pattern, while only a minority of patients presented an LQ disease.^18^

More recently, Györi *et al* described again the distribution of SLE disease activity patterns in the Hopkins Lupus Cohort, by analysing 28 years of accumulated data. The RR pattern accounted for the greatest proportion of follow-up time, being present in more than half of patient-years.^34^

As already known in the literature, the relationship between disease activity and HRQoL in SLE is controversial. HRQoL has a multifactorial origin and many factors, not always related to the disease itself, may influence the patient’s perspective on the disease.

The persistence of pain and fatigue, even when the disease is well controlled from the physician’s point of view, seems to be the most important unmet need for patients with SLE.^35^

Cross-sectional as well as longitudinal studies have clearly demonstrated that overall fatigue and disease activity follow distinct trajectories and disease activity alone cannot explain variations in fatigue over time.^36^

Patients’ degree of functioning in their daily living represents the main driver for patients’ self-evaluation of their disease status and this sometimes may lead the patients to overestimate disease activity.^11 37^

In our study cohort, fibromyalgia, irreversible organ damage and chronic glucocorticoid therapy (even at a low dosage), appears to be associated with a worse HRQoL. On the contrary, we did not find a clear association between HRQoL and disease active manifestations at the moment of evaluation.

A recent study from the longitudinal Toronto Lupus cohort showed that overall HRQoL trajectories over the 10-year study period were more likely related to the presence of fibromyalgia, with PCS having the clearest relationship. On the contrary, the association between disease activity or organ damage and PCS/MCS did not appear to be straightforward. Cumulative disease involvement of specific organ domains also did not appear to have particular relationships with PCS or MCS.^38^

Thus, our hypothesis was that the pattern of disease activity over time in SLE, more than disease activity in a single moment, could have a more significant impact on the patient’s perception.

Actually, in our cohort, we found that only a persistently long-quiescent disease can positively influence some aspects of patients’ HRQoL. In particular, compared with a RR or a CA disease, the absence of clinical manifestations of the disease for at least 3 consecutive years is associated with a better physical quality of life and minor depressive symptoms from the patient’s point of view.

We found no significant difference in PROs between CA and RR patients. This may be due to the low number of patients with CA disease. But we must also consider that patients with CA disease tended to have milder disease manifestations (eg, cutaneous or mild haematological manifestations), whereas patients with RR disease had more severe flares during the periods of activity. This study presents some limitations. First of all, we enrolled only outpatients. Moreover, the low number of patients with severe active disease, particularly with CA disease, may have hampered the possibility of making some differences emerge between the CA and RR activity patterns. However, we think that this study has some points of strength. In particular, the quite large study cohort of patients with long disease duration regularly followed at the same centre and the evaluation of different aspects of HRQoL through a wide range of validated generic and disease-specific PROs.

In summary, this study demonstrates that only a persistently quiescent disease (for at least 3 years) may improve patients’ quality of life from a physical point of view and may reduce depressive symptoms. However, a persistent condition of remission of the disease does not seem a sufficient condition to obtain a significant improvement in mental health, social functioning, fatigue and disease burden. So, these represent important unmet needs in the management of patients with such a complex disease that increase the discordance between the patient’s and the physician’s viewpoint on the disease itself. It is imperative to find a way to integrate the two perspectives into a novel strategy for the management of SLE, also relying on multidisciplinarity, non-pharmacological treatments, organisational strategies, educational activities, IT solutions to improve the communication between the parts, promoting patients’ empowerment.^39^

Therefore, a more comprehensive and holistic assessment of the patient’s condition seems necessary to set up a multitarget approach in the management of patients with SLE, with the final aim of obtaining a significant improvement in patients’ perceived well-being.

## supplementary material

10.1136/lupus-2024-001202 online supplemental file 1

10.1136/lupus-2024-001202 online supplemental file 2

## Data Availability

All data relevant to the study are included in the article or uploaded as supplementary information.
