# Supplementary material for: Impact of disease activity patterns on health-related quality of life (HRQoL) in patients with systemic lupus erythematosus (SLE)
Source: Lupus Sci Med. 2024 Jul 29;11(2):e001202. doi: 10.1136/lupus-2024-001202 (PMC11288148; doi:10.1136/lupus-2024-001202)
Supplement: online supplemental file 1 [file lupus-11-2-s001.pdf]

Table S1. Disease activity during one and three years before enrollment in chronically active and relapsing-remitting disease groups.

	Mean number of visits with active disease (± SD)	Mean total number of visits (± SD)	Mean SELENA-SLEDAI in visits with active disease (± SD)	Mean PGA in visits with active disease (± SD)
CA 1 year (34 patients)	3.03 ± 1.29	3.03 ± 1.29	4.97 ± 2.3	1 ± 0.34
CA 3 years (8 patients)	8.33 ± 3.14	8.33 ± 3.14	4.41 ± 2.19	1.06 ± 0.34
RR 1 year (53 patients)	1.33 ± 0.67	3.19 ± 0.91	5.51 ± 2.67	1.05 ± 0.4
RR 3 years (92 patients)	2.31 ± 1.53	7.38 ± 2.1	5.3 ± 2.47	1.04 ± 0.39
