# Supplementary material for: Impact of disease activity patterns on health-related quality of life (HRQoL) in patients with systemic lupus erythematosus (SLE)
Source: Lupus Sci Med. 2024 Jul 29;11(2):e001202. doi: 10.1136/lupus-2024-001202 (PMC11288148; doi:10.1136/lupus-2024-001202)
Supplement: online supplemental file 2 [file lupus-11-2-s002.pdf]

Table S2. Association between SLICC-DI and PROs, after adjusting for fibromyalgia.

	Coef.	p-value	[95% Conf. Interval]
SF-36 PCS	Coef -.04	***p*=0.000**	-.066 - .026
FACIT	Coef -.04	***p*=0.000**	-.067 - -.024
LIT	Coef .02	***p*=0.000**	.01 - .03
HADS-A	Coef .06	***p*=0.03**	.004 - .11
HADS-D	Coef .08	***p*=0.008**	.021 - .14
